# Anticipated challenges facing the dental profession: A narrative review

**DOI:** 10.1177/22799036261427949

**Published:** 2026-03-26

**Authors:** Ramya Vijeta Jathanna, Rithesh Bangera, Vinod Rakesh Jathanna

**Affiliations:** 1 Department of Orthodontics and Dentofacial Orthopedics, Manipal College of Dental Sciences, Manipal Academy of Higher Education, Manipal, India; 2 Department of Orthodontics and Dentofacial Orthopedics, KVG Dental College and Hospital, Kurunjibhag, Sullia, Karnataka, India; 3 Department of Conservative Dentistry and Endodontics, Manipal College of Dental Sciences Mangalore, Manipal Academy of Higher Education, Manipal, India

**Keywords:** dentistry, health policy, healthcare disparities, dentist practice patterns, dental technology

## Abstract

**Background:**

The dental profession is undergoing significant transformations driven by technological advancements, demographic shifts, and evolving healthcare policies. This review aimed to identify and discuss the anticipated challenges that the dental profession is likely to face in the coming decades, with a focus on technological, demographic, economic, educational, and public health aspects.

**Design and methods:**

A comprehensive literature review was conducted using databases such as PubMed, Scopus, and Google Scholar. Key search terms included “dental profession challenges,” “future of dentistry,” “dental technology,” “demographic shifts in dentistry,” and “economic impact on dental practices.” Articles were selected based on relevance, recency, and the inclusion of diverse geographic perspectives.

**Results:**

Rapid integration of digital dentistry, artificial intelligence, and tele-dentistry requires significant investment and continuous learning, posing a challenge for practitioners to stay current. An aging population and increasing demand for geriatric dental care, coupled with a workforce that is also aging, will strain resources and require specialized training.

**Conclusion:**

The dental profession faces multifaceted challenges that require coordinated efforts from educational institutions, professional organizations, policymakers, and practitioners. Addressing these challenges proactively will be essential to ensuring the sustainability and advancement of the profession.

## Introduction

The dental profession, a crucial component of global healthcare systems, is responsible for maintaining and improving oral health, which is intrinsically linked to overall well-being.^1^ Good oral health is essential not only for the prevention of dental diseases but also for avoiding complications associated with systemic conditions such as diabetes and cardiovascular diseases.^2^ The dental profession is on the cusp of significant change, influenced by advancements in digital technology, demographic shifts, and evolving healthcare policies. These dynamics are expected to impact practice models, accessibility, and the demand for specialized skills in the dental workforce. Key literature highlights trends such as digitalization and tele-dentistry and the impact of an aging population on healthcare needs.^3–5^

Despite these advancements, the dental profession is on the brink of encountering several complex challenges that threaten to disrupt the status quo. Previous research has predominantly focused on isolated aspects of these challenges, such as the integration of digital technologies into dental practice, the impact of demographic shifts on service demand, and the economic pressures on dental education and practice sustainability.^6–8^ However, there is a noticeable gap in comprehensive studies that holistically address the interconnected nature of these challenges and provide an integrated perspective on how they collectively impact the future of the profession.

This review aims to fill this gap by exploring how technological advancements, evolving patient expectations, workforce dynamics, economic pressures, and changes in education and regulatory frameworks are shaping the future of dental practice. By outlining these anticipated challenges, the review seeks to provide insights that can inform strategic planning, policy development, and professional preparedness within the field of dentistry.

As the dental profession stands at a crossroads, this study underscores the urgency of preparing for the future to continue delivering high-quality oral healthcare globally.

## Method

This qualitative, narrative review summarizes the most recent literature to comprehensively identify and analyze the anticipated challenges facing the dental profession. A comprehensive search was conducted using the following electronic databases: PubMed, Scopus, and Google Scholar. The search strategy included a combination of keywords and MeSH terms related to the dental profession and its challenges, such as “dental profession challenges,” “future of dentistry,” “dental technology,” “demographic shifts in dentistry,” “economic impact on dental practices,” “dental education,” and “public health dentistry.”

### Inclusion criteria

1. Publications addressing current or future challenges within the dental profession, including clinical, educational, technological, workforce-related, regulatory, economic, or ethical aspects.
2. Peer-reviewed journal articles, systematic reviews, narrative reviews, policy documents, expert opinion papers, professional association reports, and relevant gray literature.
3. Literature published from the year 2000 onward, considering the evolving nature of dental technologies, healthcare systems, and professional dynamics.
4. Studies published in English.
5. Full-text sources that are available through institutional access or open sources.

### Exclusion criteria

1. Publications not directly addressing challenges or future directions in the dental profession
2. Studies centered exclusively on other healthcare professions unless they contained transferable insights relevant to dentistry.
3. Articles published in languages other than English.
4. Abstract-only publications, conference posters without full reports, or sources lacking adequate methodological or contextual information.
5. Articles published before the year 2000, unless deemed seminal or foundational to understanding specific long-term trends.

This narrative review was conducted over a period of 3 months, from February 2025 to April 2025, which included literature search, screening, data extraction, and synthesis of findings.

**Figure 1.**
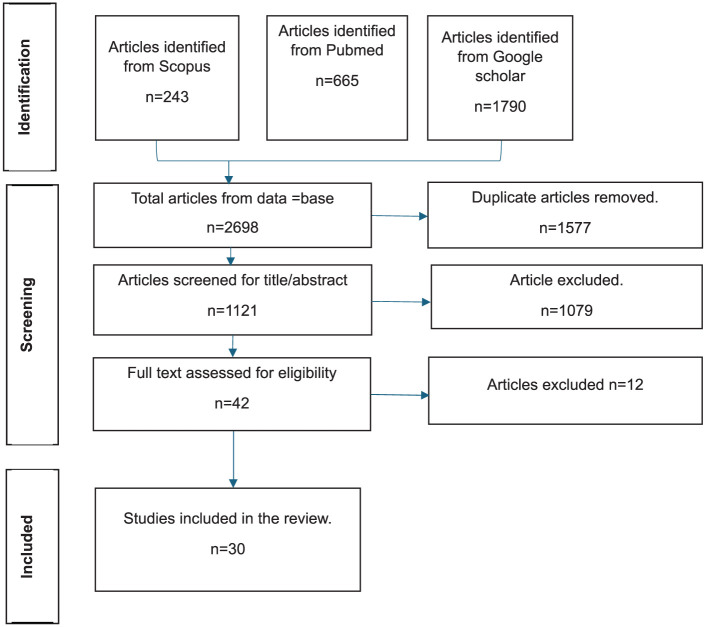
Flow diagram illustrating the selection of articles for the narrative review.

A total of 30 articles were reviewed after screening for relevance, recency, and inclusion criteria (Figure 1).

## Result and discussion

### Technological advancements

The integration of new technologies is one of the most significant challenges for dental professionals.

Innovations such as digital impressions, CAD/CAM systems, artificial intelligence (AI), and 3D printing have the potential to enhance diagnostic accuracy, improve treatment outcomes, and streamline clinical workflows. However, their adoption requires continuous investment in equipment, training, and practice redesign.

Digital dentistry encompasses a wide range of tools that improve precision and efficiency. CAD/CAM technology, for example, reduces fabrication time while providing highly accurate and well-fitting restorations, making it an increasingly preferred option for clinicians seeking to enhance treatment outcomes.^9,10^

Similarly, digital impression systems use intraoral scanners offer superior accuracy compared to traditional methods, improve patient comfort and facilitate faster turnaround times for dental prosthetics and reduce the risk of errors during the impression-taking process.^11^

3D printing technology is transforming restorative and orthodontic practice through the production of customized appliances, including surgical guides, aligners, and implants. Its capacity for high precision and rapid prototyping allows dental professionals to fabricate patient-specific devices with improved accuracy and reduced production time.^12^ AI has emerged as a powerful tool across diagnostics, treatment planning, and administrative workflow. AI-enabled systems can interpret radiographic images with high accuracy, detect early signs of caries or periodontal disease, and support clinicians in developing individualized treatment plans derived from large datasets.^13,14^ AI can streamline administrative tasks such as appointment scheduling, patient reminders, and billing. Additionally, AI-driven virtual assistants can provide patients with information and answer common queries, enhancing the overall patient experience.^15^

Tele-dentistry, as a complementary digital tool, enables remote consultations and follow-up care, improving access for patients in rural or mobility-restricted settings. Evidence suggests that it is particularly useful for routine monitoring and triage, although its role must be integrated thoughtfully within broader care pathways.^16^

Despite the potential benefits, the adoption of these technological advancements presents several challenges. The initial investment required for acquiring and implementing advanced technologies such as CAD/CAM systems, digital scanners, and AI software can be substantial. Small and mid-sized dental practices may find these costs prohibitive, limiting widespread adoption.^9,10^ Effective use of new technologies necessitates comprehensive training for dental professionals. Continuous education and skill development are essential to keep pace with technological advancements, which can be time-consuming and costly.^17^ Integrating new tools into established clinical workflows may require significant operational restructuring. Additionally, digital systems raise concerns regarding patient privacy, data security, and regulatory compliance. Addressing these challenges is essential to ensure safe and effective implementation of emerging dental technologies.

### Demographic shifts

The dental profession is increasingly influenced by significant demographic changes, which present unique challenges and opportunities. Key demographic trends include an aging population, shifts in workforce demographics, and the effects of migration and mobility.

An aging global population increases the demand for specialized geriatric dental care, as older adults often present with complex oral needs, multiple comorbidities, and accessibility barriers.^18^ At the same time, the dental workforce itself is aging, with many practitioners nearing retirement, raising concerns about workforce shortages and the loss of clinical expertise and mentorship.^19^ Attracting new graduates to replace retiring dentists is crucial. This involves not only ensuring that dental education programs produce enough graduates but also making the profession appealing through competitive compensation, work-life balance, and opportunities for professional growth.

Global migration and internal mobility trends affect the distribution of dental professionals and the demand for dental services. Migration can lead to population growth in certain areas, increasing the demand for dental services. Conversely, rural and underserved areas may experience a decline in population, exacerbating existing disparities in access to care.^20^ Migration contributes to greater ethnic and cultural diversity within patient populations. Dental professionals must be culturally competent and able to provide care that respects and understands different cultural practices and oral health beliefs.

The combined effects of an aging workforce and increased demand for services due to demographic changes may lead to significant workforce shortages. This is particularly acute in rural and underserved areas, where recruiting and retaining dental professionals is already challenging. Addressing these demographic trends requires targeted policies, incentives for rural practice, and enhanced training in geriatric dentistry, cultural competency, and the management of medically complex patients. Governments and professional bodies must also invest in education, continuing professional development, and supportive practice models to ensure the profession can meet evolving population needs.^21^

Policies and incentives are needed to encourage dental professionals to practice in underserved areas, such as loan repayment programs, scholarships, and higher reimbursement rates for services provided in these locations.

### Economic pressures

Economic pressures represent a significant challenge for the dental profession, affecting both dental education and practice sustainability. Key economic factors collectively impact the affordability of dental care, the economic viability of dental practices, and efforts to address health disparities.

The cost of dental education is a critical issue facing aspiring dental professionals. High tuition fees, combined with the costs of books, equipment, and living expenses, result in significant debt for many dental students upon graduation. This debt can influence career choices, with graduates potentially prioritizing higher-paying specialties or practice settings over general practice or service in underserved areas exacerbating workforce shortages.^22–24^ It can also affect the demographic diversity of dental professionals, as individuals from lower socioeconomic backgrounds may find it challenging to afford the education required to enter the profession.

Operating a dental practice is becoming increasingly costly due to the need to invest in new technologies such as CAD/CAM systems, digital imaging, and electronic health records, all of which require substantial upfront and maintenance expenses.^25^ Rising and fluctuating material costs, influenced by global supply chains, further affect financial stability. Additionally, compliance with infection control standards, data protection regulations, and occupational safety requirements, while essential for maintaining high-quality care, adds to the overall operational burden.^26^ Healthcare funding, including dental insurance coverage and public funding for dental services, varies widely across regions, affecting access to and affordability of care. Dental insurance coverage is not as comprehensive as medical insurance in many countries. Coverage often focuses on preventive and basic restorative services, with limited benefits for more complex procedures. This variability can lead to significant out-of-pocket expenses for patients, reducing their ability to afford necessary dental care.^27^

In some regions, public funding for dental services is limited or non-existent. This lack of funding creates disparities in access to care, particularly for low-income populations and those in rural or underserved areas. Public health programs that provide dental services to vulnerable populations are crucial but are often underfunded and unable to meet the demand.^28^

One of the primary challenges is making dental care affordable for all patients. Strategies to address this include expanding dental insurance coverage, increasing public funding for dental services, and implementing sliding scale fees based on patients’ ability to pay.

Economic pressures contribute to health disparities in dental care, particularly for low-income populations and rural population who often face barriers to accessing care due to cost and lack of providers. Policies and programs aimed at reducing these disparities are essential. This includes incentivizing dental professionals to work in underserved areas through loan repayment programs and scholarships, as well as increasing funding for public health dental programs.

At practice level, adopting technologies that enhance productivity and reduce long-term costs, such as digital dentistry tools and practice management software can help mitigate some economic strain and support long term sustainability

### Educational and training needs

The evolving landscape of the dental profession demands significant reforms in education and training to prepare practitioners for future challenges.

Dental education must integrate training on advanced technologies such as CAD/CAM systems, digital imaging, and 3D printing. Students should gain hands-on experience with these tools to develop the skills necessary for modern dental practice.^29^ The curriculum should emphasize interdisciplinary collaboration, recognizing the connection between oral health and overall health.^30^ This includes training on how to work with other healthcare professionals to manage conditions like diabetes and cardiovascular diseases, which have oral health implications. The majority of colleges lack training in specialized areas such as geriatrics, pediatrics, compromised cases, and community-based training.^23^ The lack of infrastructure in many colleges could be due to the tremendous financial burden on the institutions. A modified dental curriculum closing all the evidence-based gaps in dental education is required. Emphasizing patient-centered care and communication skills is also crucial. Dental professionals need to be adept at understanding patient needs, preferences, and cultural backgrounds to provide effective care.

The fast pace of technological and clinical advancements in dentistry necessitates continuous professional development. Continuing education programs should be readily available and cover a wide range of topics, including new technologies, updated clinical guidelines, and emerging research findings. Online courses, webinars, and regional workshops can provide flexible learning opportunities that accommodate different schedules and geographic locations.

Strategies to encourage dental professionals to practice in rural and underserved areas include financial incentives, such as loan repayment programs, scholarships, and grants. These incentives can make working in these areas more attractive to new graduates.^31^

Incorporating community-based education and service-learning experiences into the dental curriculum can expose students to the needs of underserved populations and inspire them to consider careers in these areas. Providing robust support systems, such as mentoring programs and professional networks, can help retain dental professionals in underserved areas. Support can mitigate feelings of isolation and professional burnout, which are common in these settings.

Creating effective incentives to attract dental professionals to underserved areas is challenging. Policymakers and educational institutions must collaborate to develop and implement programs that offer meaningful benefits and address the specific needs of these regions.^32^

Dental education must also address the need for cultural competence and diversity within the profession. Training programs should include components that prepare students to work with diverse patient populations and understand the social determinants of health.

### Public health concerns

Public health concerns significantly impact on the dental profession, shaping how dental care is delivered and integrated within the broader healthcare system. Chronic diseases such as diabetes, cardiovascular diseases, and other systemic conditions have a profound impact on oral health.

Diabetes is closely linked with periodontal disease, with a bidirectional relationship where each condition can exacerbate the other. Diabetic patients are at a higher risk of developing severe gum disease, which can lead to tooth loss and complicate diabetes management.^33^ Effective management of oral health in diabetic patients is crucial for controlling blood sugar levels and preventing complications.^34^

Poor oral health, particularly periodontal disease, has been associated with an increased risk of cardiovascular diseases, including heart disease and stroke. Inflammation and infections in the oral cavity can contribute to systemic inflammation, affecting cardiovascular health. Dental professionals play a vital role in identifying and managing oral conditions that may impact cardiovascular health.^35^ Chronic conditions such as osteoporosis, respiratory diseases, and autoimmune disorders can also impact oral health. Medications used to manage these diseases often have side effects that affect the oral cavity, such as dry mouth and changes in the oral microbiome. Dental professionals need to be aware of these interactions and manage them effectively.^36^

Preventive care is a cornerstone of public health dentistry, emphasizing the importance of early intervention and maintenance to prevent dental diseases. Preventive strategies include regular dental check-ups, patient education on oral hygiene practices, fluoride treatments, and dental sealants. These measures help reduce the incidence of dental caries, gum disease, and other oral health issues, leading to better overall health outcomes.

Public health initiatives such as community water fluoridation and school-based dental programs are essential for reaching broader populations, particularly underserved communities. These programs promote oral health awareness and provide preventive services to those who may not have access to regular dental care.

The integration of dental and medical care is essential for comprehensive health management, ensuring that oral health is not treated in isolation from overall health. Effective communication and collaboration between dental and medical professionals can enhance patient care. Integrated care models, where dental and medical records are shared, and multidisciplinary teams work together, can improve the management of conditions that affect both oral and systemic health.

Viewing oral health as an integral part of overall health encourages holistic approaches to patient care. Dental professionals should be involved in general health screenings and referrals, while medical professionals should consider oral health in their diagnoses and treatment plans. Educating patients about the links between oral health and systemic health is crucial. Empowering patients to take an active role in managing their oral health can lead to better health behaviors and outcomes.

Addressing public health concerns in dentistry involves overcoming several challenges to ensure comprehensive and equitable care. Social determinants such as socioeconomic status, education, and access to healthcare significantly impact oral health outcomes. Dental professionals and public health policymakers must work together to address these determinants by implementing policies and programs that reduce barriers to care, such as expanding access to affordable dental services and increasing public health funding.

Disease patterns are changing, with an increase in chronic diseases and their impact on oral health. Dental professionals must stay informed about these trends and adapt their practices to manage new challenges effectively. This includes continuing education and training in managing the oral health implications of chronic diseases and evolving public health threats.

### Regulatory and policy issues

The dental profession operates within a complex framework of regulatory and policy issues that ensure the provision of safe and effective care.

Licensing and accreditation are fundamental mechanisms that ensure consistent standards of dental care across different regions. Licensing requirements for dental professionals vary by country and region, but they generally include educational prerequisites, examinations, and continuing education mandates. Accreditation of dental schools and programs ensures that they meet established standards of education, preparing students for competent practice. Consistency in these standards is essential for maintaining public trust and ensuring that dental professionals possess the necessary skills and knowledge.^37^ Efforts to harmonize licensing and accreditation standards internationally can facilitate the mobility of dental professionals and enhance global health outcomes. This includes mutual recognition agreements between countries and the development of international accreditation standards.

The scope of practice for dental professionals is evolving, particularly with the inclusion of dental auxiliaries and mid-level providers. These professionals can perform a range of preventive and restorative procedures, often under the supervision of a dentist. This expansion aims to increase access to care, particularly in underserved areas.^38^

Changes in legislation and regulations are necessary to define and authorize the expanded scope of practice for these professionals. International organizations, such as the World Health Organization (WHO), develop guidelines and agreements that influence national health policies, including oral health strategies. These initiatives often aim to address global health challenges such as non-communicable diseases, which include oral health conditions. Adapting international guidelines to local contexts requires collaboration between policymakers, dental associations, and healthcare providers. This ensures that global recommendations are effectively integrated into national health systems and address the specific needs of local populations.

Regulatory environments are constantly evolving, and dental professionals must stay informed about changes that affect their practice. This includes new regulations related to infection control, data protection, and clinical procedures. Adapting to these changes requires ongoing education and compliance efforts. While international guidelines aim to standardize care and improve health outcomes globally, aligning these with local regulations and practices can be challenging. Differences in healthcare infrastructure, economic conditions, and cultural practices must be considered when implementing global standards locally.

Ethical considerations are paramount in the dental profession, particularly in the context of new technologies and expanding scopes of practice. Issues such as patient consent, privacy, and equitable access to care must be addressed. Dental professionals must navigate these ethical dilemmas while adhering to regulatory requirements and professional standards.

Dental professionals and organizations play a crucial role in advocating policies that support the profession and public health. This includes participating in policy development processes, engaging with stakeholders, and promoting research to inform evidence-based regulations.^39^

Ensuring that dental regulatory bodies and educational institutions have sufficient funding and resources is critical for maintaining high standards. This includes investment in research, technology, and infrastructure to support regulatory compliance and the continuous improvement of dental education and practice.

### Limitations

The review is not a systematic synthesis and therefore is subjected to selection bias because article inclusion relied on author judgment rather than a predefined protocol. The search strategy, although comprehensive, may not have captured all relevant literature, particularly unpublished studies or gray literature not indexed in major databases. The absence of quantitative synthesis limits the ability to compare findings across studies. Additionally, the broad scope of the review introduces heterogeneity in the evidence, making direct comparison challenging. Finally, because developments in dental technology and policy evolve rapidly, some insights may become outdated as newer trends emerge.

## Conclusion

The dental profession faces a myriad of challenges that span technological advancements, demographic shifts, economic pressures, educational and training needs, public health concerns, and regulatory and policy issues. Addressing these challenges is crucial for the continued provision of high-quality dental care and the overall health of the population.

Future research should focus on evaluating the effectiveness of new technologies and educational approaches, understanding the impact of demographic changes on dental care demand, and exploring innovative models of care delivery. Policy development should prioritize creating an enabling environment for the integration of dental and medical care, addressing workforce maldistribution, and ensuring equitable access to care.

The dental profession must navigate a complex landscape of challenges that require coordinated efforts, strategic planning, and a commitment to continuous improvement. By embracing innovation, fostering collaboration, and prioritizing research and policy development, the dental profession can overcome these challenges and continue to advance oral health globally.
